# ALKBH5 Regulates SPHK1-Dependent Endothelial Cell Angiogenesis Following Ischemic Stress

**DOI:** 10.3389/fcvm.2021.817304

**Published:** 2022-01-20

**Authors:** Rajesh Kumari, Roshan Dutta, Prabhat Ranjan, Zainab Gbongbo Suleiman, Sumanta Kumar Goswami, Jing Li, Harish Chandra Pal, Suresh Kumar Verma

**Affiliations:** ^1^Division of Cardiovascular Disease, Department of Medicine, The University of Alabama at Birmingham, Birmingham, AL, United States; ^2^Department of Biomedical Engineering, The University of Alabama at Birmingham, Birmingham, AL, United States; ^3^Department of Pathology, Molecular and Cellular Pathology, The University of Alabama at Birmingham, Birmingham, AL, United States

**Keywords:** angiogenesis, ischemia, RNA demethylase, SPHK1, m6A RNA methylation, endothelia cell

## Abstract

**Background:**

Endothelial cells dysfunction has been reported in many heart diseases including acute myocardial infarction, and atherosclerosis. The molecular mechanism for endothelial dysfunction in the heart is still not clearly understood. We aimed to study the role of m^6^A RNA demethylase alkB homolog 5 (ALKBH5) in ECs angiogenesis during ischemic injury.

**Methods and Results:**

ECs were treated with ischemic insults (lipopolysaccharide and 1% hypoxia) to determine the role of ALKBH5 in ECs angiogenesis. siRNA mediated ALKBH5 gene silencing was used for examining the loss of function. In this study, we report that ALKBH5 levels are upregulated following ischemia and are associated with maintaining ischemia-induced ECs angiogenesis. To decipher the mechanism of action, we found that ALKBH5 is required to maintain eNOS phosphorylation and SPHK1 protein levels. ALKBH5 silencing alone or with ischemic stress significantly increased SPHK1 m^6^A mRNA methylation. In contrast, METTL3 (RNA methyltransferase) overexpression resulted in the reduced expression of SPHK1.

**Conclusion:**

We reported that ALKBH5 helps in the maintenance of angiogenesis in endothelial cells following acute ischemic stress via reduced SPHK1 m^6^A methylation and downstream eNOS-AKT signaling.

## Introduction

Cardiovascular diseases (CVDs) including coronary artery disease, cardiac fibrosis and hypertrophy are the leading cause of deaths world-wide (1, 2). The physiology of the heart is regulated by multiple factors, including endothelial vascular integrity, endocrine, paracrine and autocrine signaling and epigenetics (3–6). Endothelial cells (ECs) dysfunction and its role in promoting coronary artery disease is well-characterized. During chronic heart failure, endothelial nitric oxide synthase (eNOS) mediated production of nitric oxide (NO) is impaired along with migration and repair capacity of endothelial progenitor cells (7–9). A wide variety of kinases such as sphingosine kinase-1 (SPHK1), regulate multi-site eNOS phosphorylation, which ultimately regulates its subcellular localization and protein-protein interactions (10–12). SPHK1 phosphorylates sphingosine-1, a key extracellular and intracellular messenger which regulates multiple aspects of vascular biology and physiology (13).

Current advancements in epigenetic research, including epitranscriptomic regulation, suggested its role in regulating cardiovascular functions and kindled the hopes for effective RNA-based therapeutics to manage CVD (14, 15). Recent studies have improved our understanding of N^6^-methyladenosine (m^6^A) mRNA methylation and its functional importance in RNA stability, translation efficiency, splicing, processing and degradation (16–21). The m^6^A methylation is catalyzed by methyltransferase complex known as “m^6^A writers” having methyltransferase like 3 (METTL3) protein as a key enzyme with methyl group transferase activity. After the addition of m^6^A, either it will be recognized by “m^6^A reader” which include proteins of YTHDF (YTH domain-containing m^6^A RNA Binding Proteins) family or erased with “m^6^A erasers” like alkB homolog 5 (ALKBH5) or fat mass and obesity-associated protein (FTO) to perform cellular functions (22–28).

The significant research has been carried out to understand the role of m^6^A RNA methylation in CVDs. Dorn et al., revealed the role of METTL3 in regulating compensated cardiomyocyte hypertrophy which significantly reduced cardiac output in age-related manner; in contrast, Kmietczyk et al. suggested that METTL3 overexpression leads to pathological hypertrophy (29, 30). Furthermore, Berulova et al. showed that m^6^A methylation regulates the stability of mRNAs associated with metabolic and other regulatory pathways during heart failure (31). Interestingly, a negative feedback loop between METTL3 and ALKBH5 is reported in cardiomyocytes following hypoxia/reoxygenation and showed that it inhibits autophagy via destabilizing tfeb mRNA, a master regulator of lysosomal biogenesis and autophagy genes (32).

Previously, reduced expression of ALKBH5 was shown in the murine hearts in the first week of ischemic injury but was recovered at 4 weeks (33). However, the role of ALKBH5 in ECs biology and function is not studied. As early ischemic injury-induced angiogenesis post-myocardial infarction (34), we hypothesized that short-term ischemic stimuli activate ALKBH5 which helps in the maintenance of endothelial cell angiogenesis. In fact, hypoxia alters the expression of m^6^A eraser ALKBH5 *via* hypoxia-inducible factor (HIF) 1α which is regulated by SPHK1 (35, 36). Here, we reported that the silencing of ALKBH5 results in impaired endothelial tube formation following ischemia. Further, we demonstrated that ALKBH5 maintains SPHK1 protein level and eNOS phosphorylation (which is required to regulate EC tube formation) following ischemia in ECs. Interestingly, ALKBH5 mediated maintenance of angiogenesis is vascular endothelial growth factor A (VEGF-A)-independent.

## Results

### ALKBH5 Expression Was Increased in Endothelial Cells Under Hypoxia and LPS Incubation

**Figure 1:**
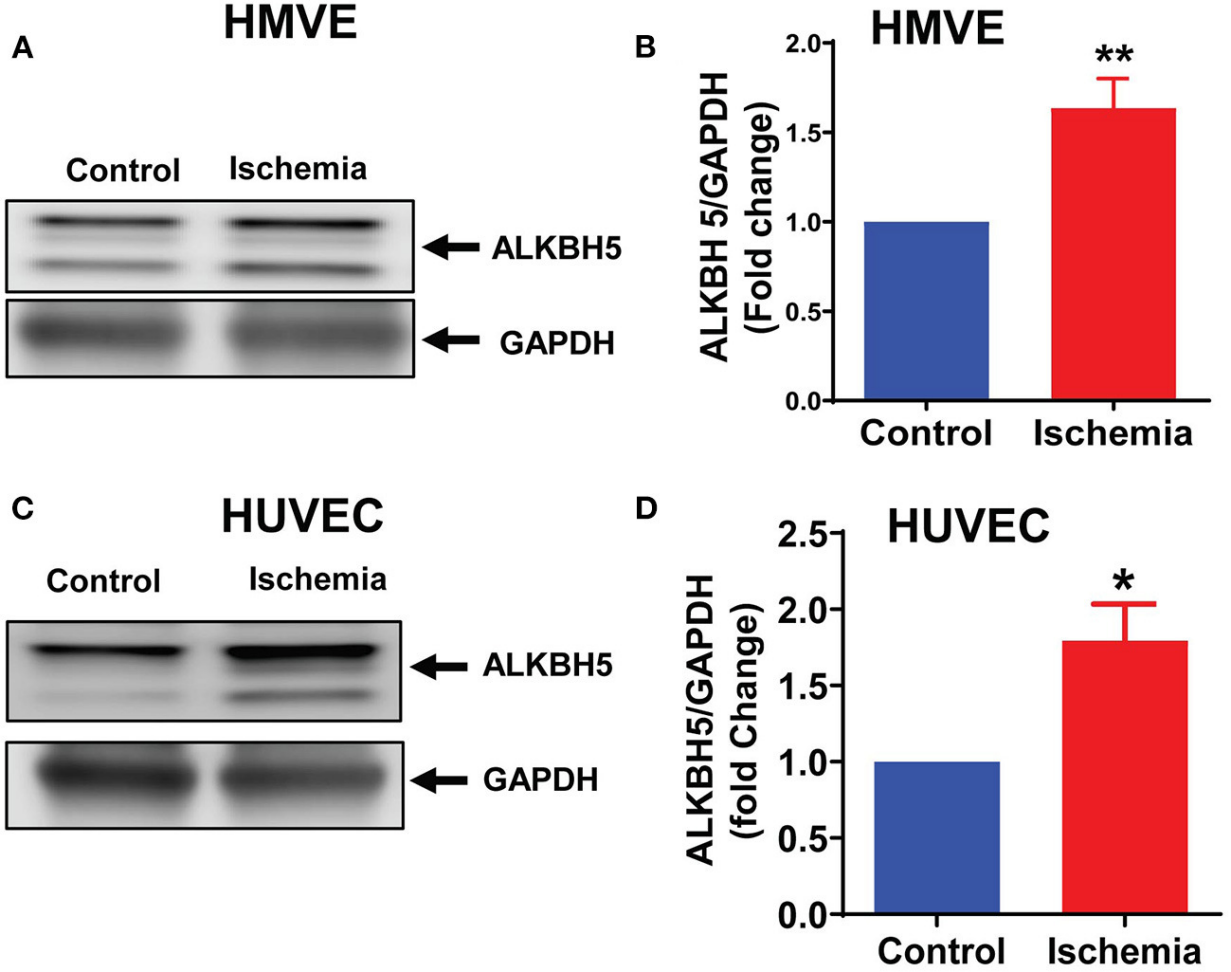
Ischemic stress increased ALKBH5 expression. HMVE and HUVEC cells were treated with ischemic stress for 24 h. Proteins were isolated for western blotting (WB). WB is showing protein expression of ALKBH5 in HMVE **(A,B)**, HUVEC **(C,D)**. Increased AKLBH5 expression was observed following ischemic stress in both human endothelial cells lines. **P < 0.01, control vs. 24 h ischemia, *P < 0.05, control vs. 24 h ischemia (*n* = 3–5).

To measure the effect of ischemic injury on the ALKBH5 expression, we cultured human ECs (HMVE and HUVEC) under ischemic stress as described in method section for 24 h. ALKBH5 expression was significantly increased in the human ECs (Figures 1A–D). Our data suggest that acute ischemic stress (nutrient deprivation together with hypoxia and LPS) significantly induced ALKBH5 expression in endothelial cells.

### Downregulation of ALKBH5 Results in Impaired Endothelial Cell Angiogenesis

**Figure 2:**
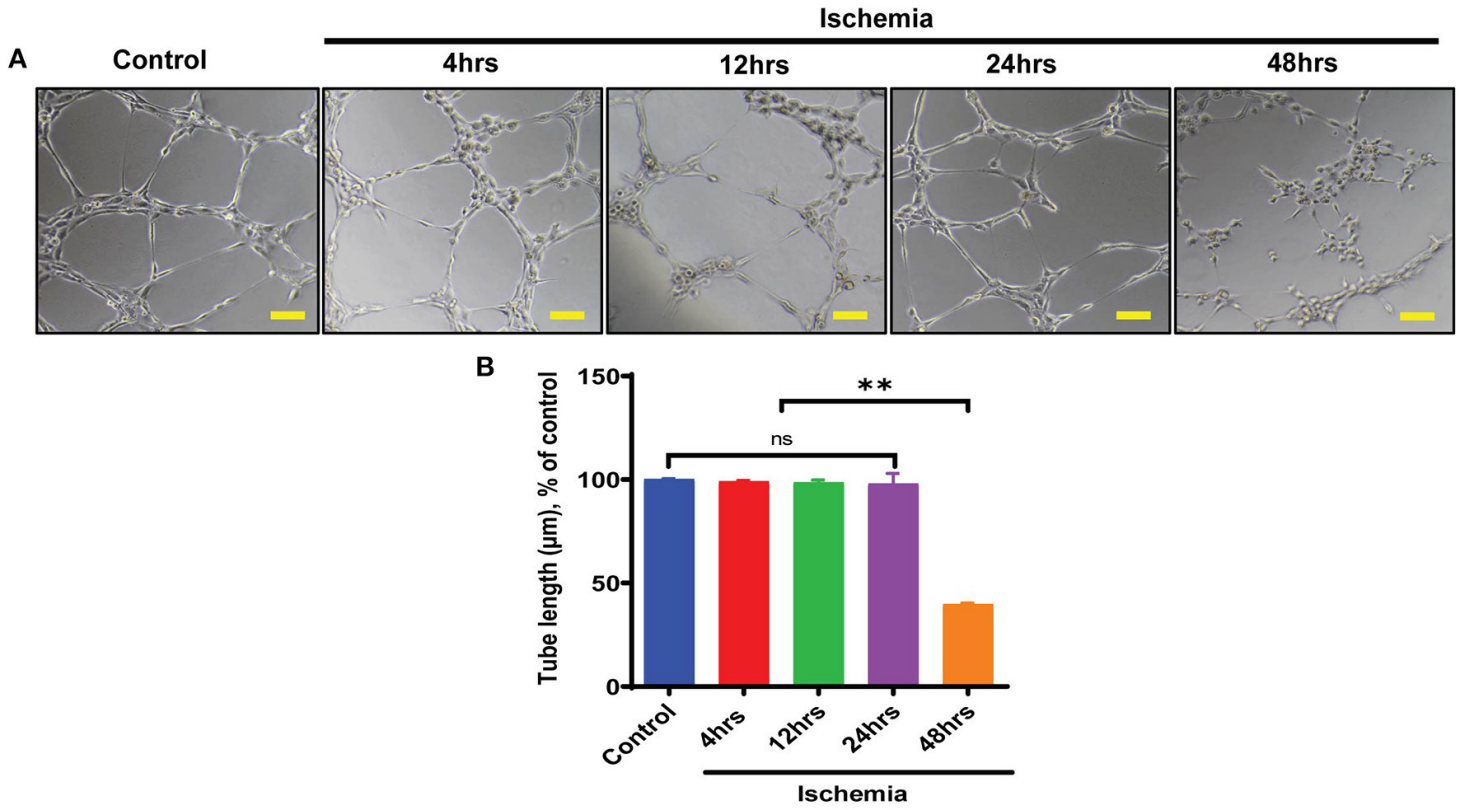
Endothelial cell tube formation was measured following ischemic stress. **(A)** HMVE were cultured under ischemia for indicated time points in low serum media. For the tube formation analysis, cells were trypsinized and seeded on matrigel. **(B)** The images were taken at 100X magnification and tube length was measured with ImageJ (NIH) software (total 40–50 branch points were measured to take the average for each specimen).The scale bar on Image represents 50 μM pixel size. **P < 0.01, control vs. 48 h ischemic stress. ns indicates not significant (*n* = 3–5).

**Figure 3:**
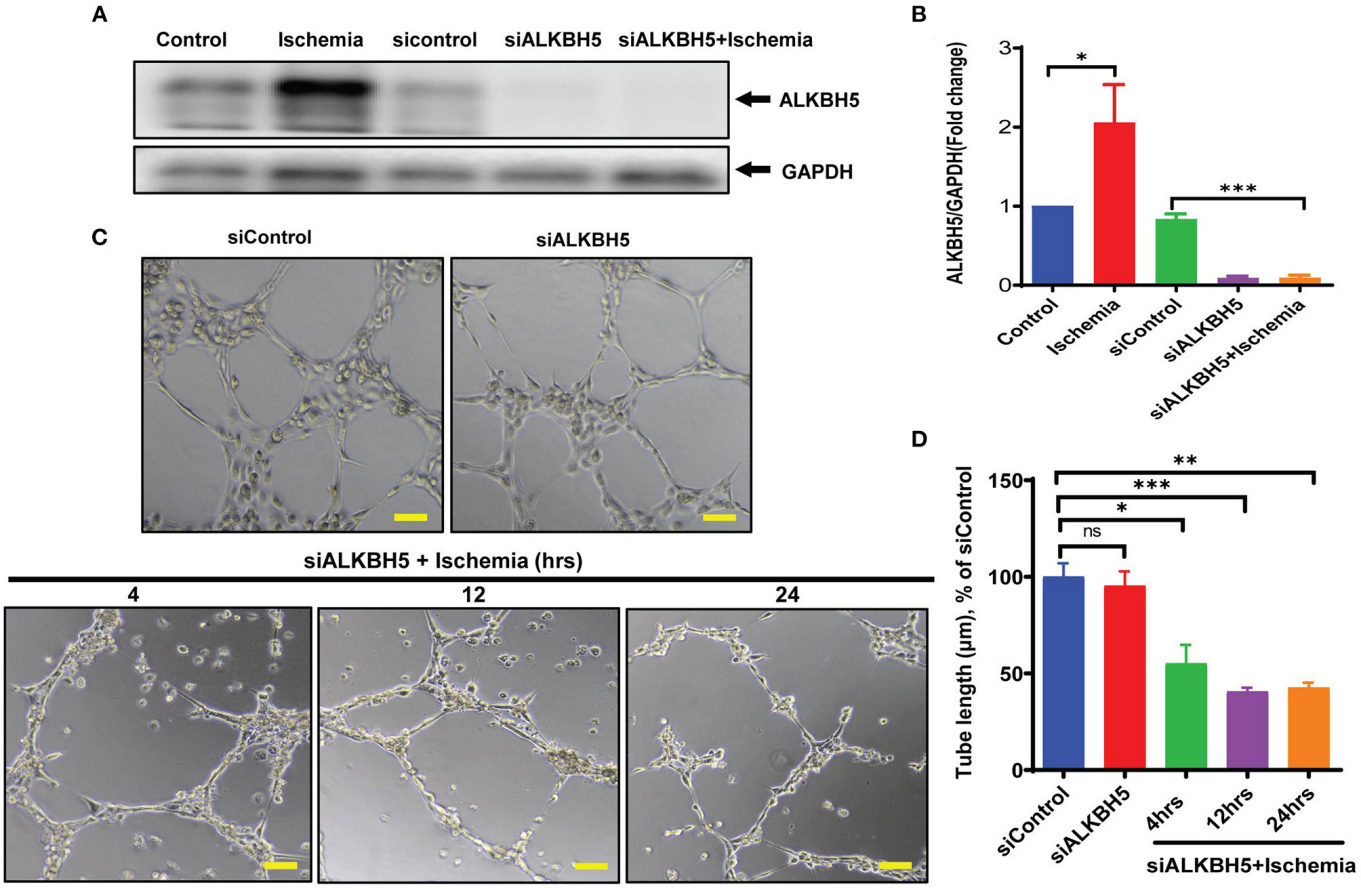
ALKBH5 silencing impaired the tube formation in ECs. **(A)** HMVE cells were transfected with 10 nM siALKBH5 siRNA for 18 h before ischemic stimulus. Scrambled RNA was used as a negative control. **(B)** WB quantification revealed an increased expression of ALKBH5 following treatments. ALKBH5 silencing inhibits ischemic stress induced ALKBH5 expression. *P < 0.05, control vs. 24 h ischemic stress; ***P < 0.001, siRNA negative control vs. siALKBH5; **(C)** HUVEC cells were treated with ischemic stress following ALKBH5 silencing. Cells were trypsinized and seeded on matrigel. **(D)** Tube formation was observed under the microscope at 100X magnification and tube length was measured with ImageJ (NIH) software (total 40–50 branch points were measured to take the average for each specimen). The scale bar on Image represents 50 μM pixel size. *P < 0.05, siRNA negative control vs. siALKBH5 with 4 h ischemia; ***P < 0.001, siRNA negative control vs. siALKBH5 with 12 h ischemia; **P < 0.01, siRNA negative control vs. siALKBH5 with 24 h ischemia; ns indicates not significant (*n* = 3–5).

As along with cardiomyocytes and fibroblasts, ECs are a major constitutional component of the heart and help in the maintenance of oxygen and nutrients supply to the heart by forming blood vessels. Next, we aimed to study the angiogenic potential of human endothelial cells following 4–48 h of ischemic stress. Matrigel tube formation analysis suggests that ECs angiogenesis was preserved till 24 h ischemia (Figures 2A,B). In contrast, chronic long-term (48 h) ischemia significantly impaired endothelial cells tube formation (Figures 2A,B and Supplementary Figures 1A,B). Further, to determine whether ALKBH5 plays important role in the maintenance of tube formation following acute ischemic injury (24 h or before), first we inhibited ALKBH5 using ALKBH5 siRNA in EC and then stimulated ischemic stress for 24 h. ALKBH5 inhibition efficiently reduced ALKBH5 protein expression (Supplementary Figures 3A–D). Interestingly, ALKBH5 silencing significantly reduced ischemia-induced ALKBH5 protein expression (Figures 3A,B). Further, ALKBH5 silencing significantly impaired ischemia-induced tube formation at all time-points from 4 to 24 h (Figure 3D and Supplementary Figures 1C,D). As ECs have immense potential to migrate, next we measured the effect of ALKBH5 inhibition on EC migration. In corroboration with our tube formation data, ischemic stress induced EC migration was significantly reduced following ALKBH5 silencing (Supplementary Figures 2A,B). Interestingly, ALKBH5 silencing alone did not alter ECs tube formation (Figures 3C,D and Supplementary Figures 2A,B). These data confirm that ALKBH5 is required in the maintenance of angiogenesis only during ischemic stress. Further, to rule out the possibility, whether impaired angiogenesis is due to increased ECs cell death, next we performed TUNEL and Annexin V staining. ALKBH5 silencing alone or together with ischemic stress (24 h) did not alter EC death (Supplementary Figures 4A–C). Our data confirms that ALKBH5 helps in the maintenance of ECs angiogenesis only during acute ischemic stress.

### ALKBH5 Regulates eNOS Phosphorylation *via* Regulating AKT Phosphorylation

**Figure 4:**
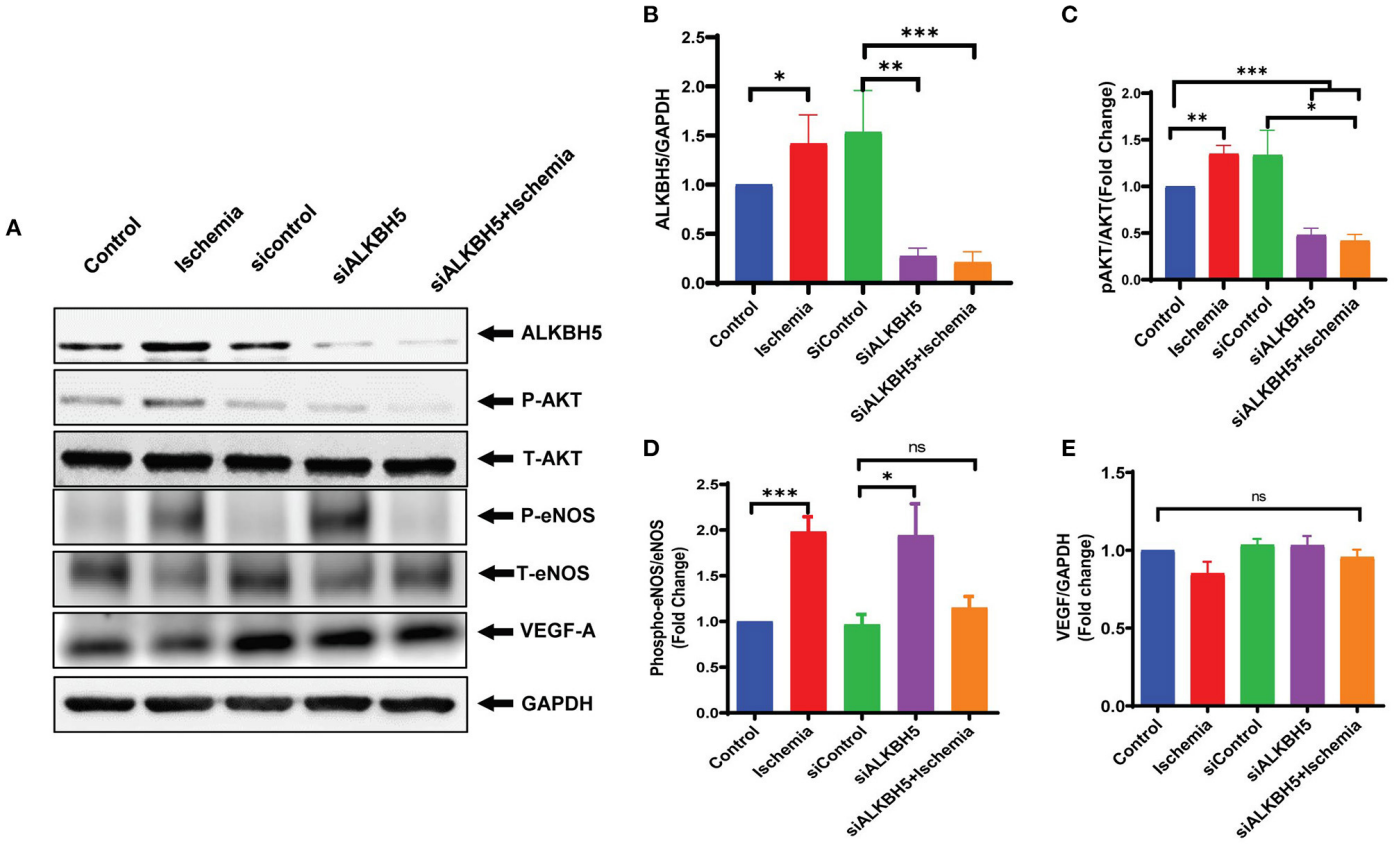
Measurement of endothelial angiogenesis markers following ischemic stimulation. HMVE cells were cultured under ischemia for 24 h. **(A)** Western blot showing expression of ALKBH5, Phospho AKT, Total AKT, phospho-eNOS, total eNOS and VEGF-A. **(B)** Quantification of ALKBH5 vs GAPDH. **(C)** Quantification for Phospho-Akt vs Total-AKT level. **(D)** Quantification of Phospho-eNOS vs Total-eNOS. **(E)** Quantification of VEGF-A vs GAPDH. Ischemic stress after ALKBH5 silencing significantly increased AKT and eNOS phosphorylation but the expression of VEGF-A was not changed. *P < 0.05, **P < 0.01, ***P < 0.001, ns indicates not significant with respective control (*n* = 3–5).

Previous studies showed the important role of eNOS and VEGF-A signaling mechanism in the angiogenesis process in heart (37–39). The eNOS phosphorylation is required for the synthesis of nitric oxide (NO). NO is a key signaling molecule and regulates EC functions (40, 41). AKT promotes eNOS phosphorylation and AKT/eNOS axis is well-established to regulate angiogenesis and growth hormone signaling in endothelial cell (42, 43). To ascertain the mechanism by which ALKBH5 helps in the maintenance of EC angiogenesis, next we measured AKT and eNOS phosphorylation, and VEGF-A protein expression following ALKBH5 silencing and ischemic stress. Ischemic stress significantly increased both AKT and eNOS phosphorylation (Figures 2A,B and Supplementary Figures 2A,B). Interestingly, ALKBH5 silencing (Figures 4A,B) significantly reduced ischemia induced AKT and eNOS phosphorylation (Figures 4A,C,D). For our surprise, neither ALKBH5 silencing nor ischemic stress altered VEGF-A expression (Figures 4A,E). Overall, these data suggest that ALKBH5 maintains EC angiogenesis by regulating AKT and eNOS phosphorylation and it is independent of VEGF-A signaling.

### ALKBH5 Regulates m^6^A Abundance on SPHK1 Transcript

**Figure 5:**
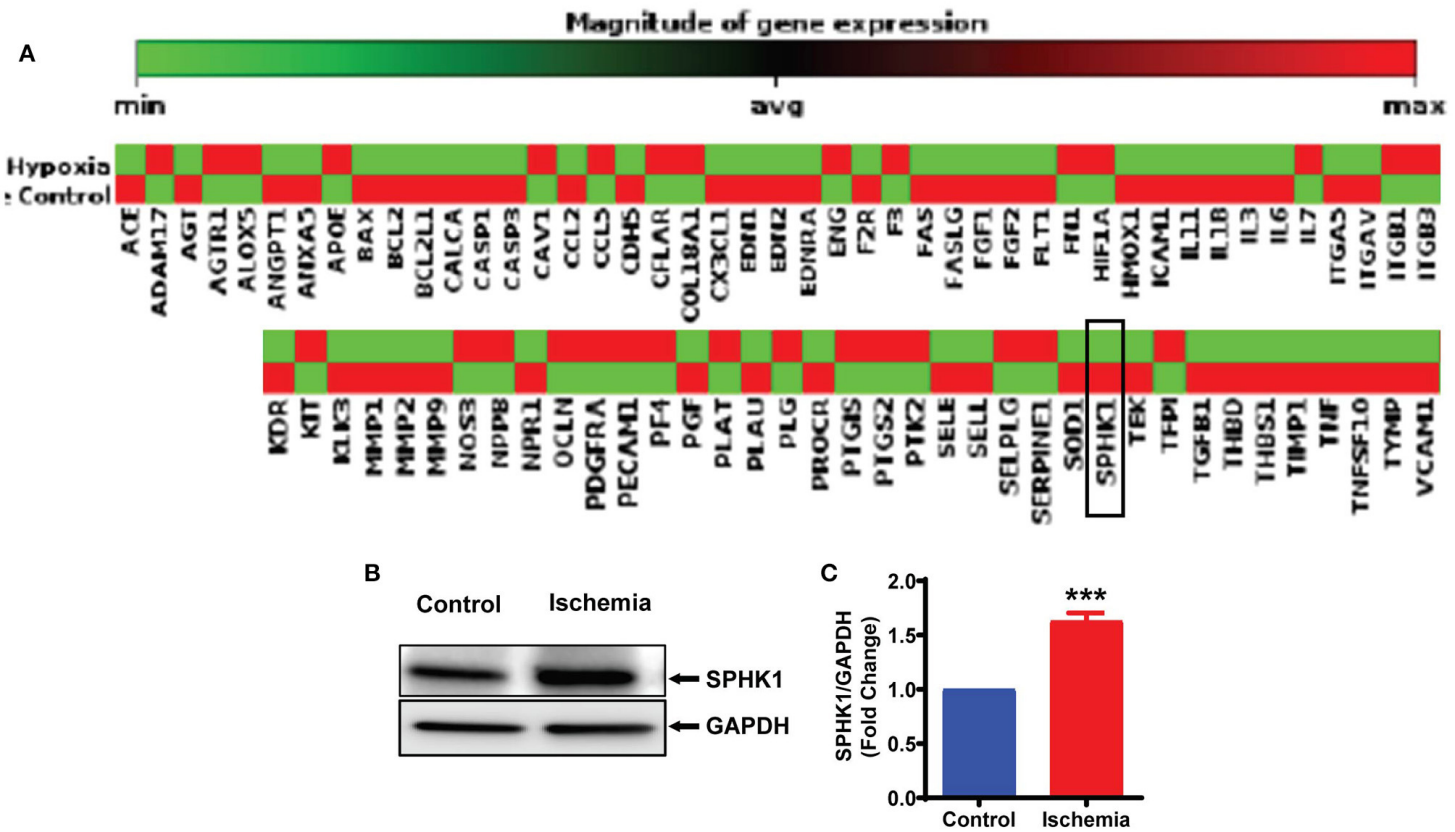
Ischemia-induced SPHK1 expression in endothelial cells. **(A)** HMVE cells were cultured under hypoxia (under 1% O_2_ levels) and mRNA was isolated for RT^2^ profiler PCR array for genes implicated in endothelial cell biology. SPHK1 gene expression was downregulated following hypoxia. **(B)** HMVE cells were cultured under LPS with hypoxia for 24 h and protein lysates were prepared for western blotting. **(C)** SPHK1 protein was significantly upregulated by LPS with hypoxia treatment. SPHK1 bands intensity were quantified using ImageJ (NIH) from 3 independent experiments. Data represented as means ± SEM. ***P < 0.001, control vs. 24 h ischemia (*n* = 3–5).

**Figure 6:**
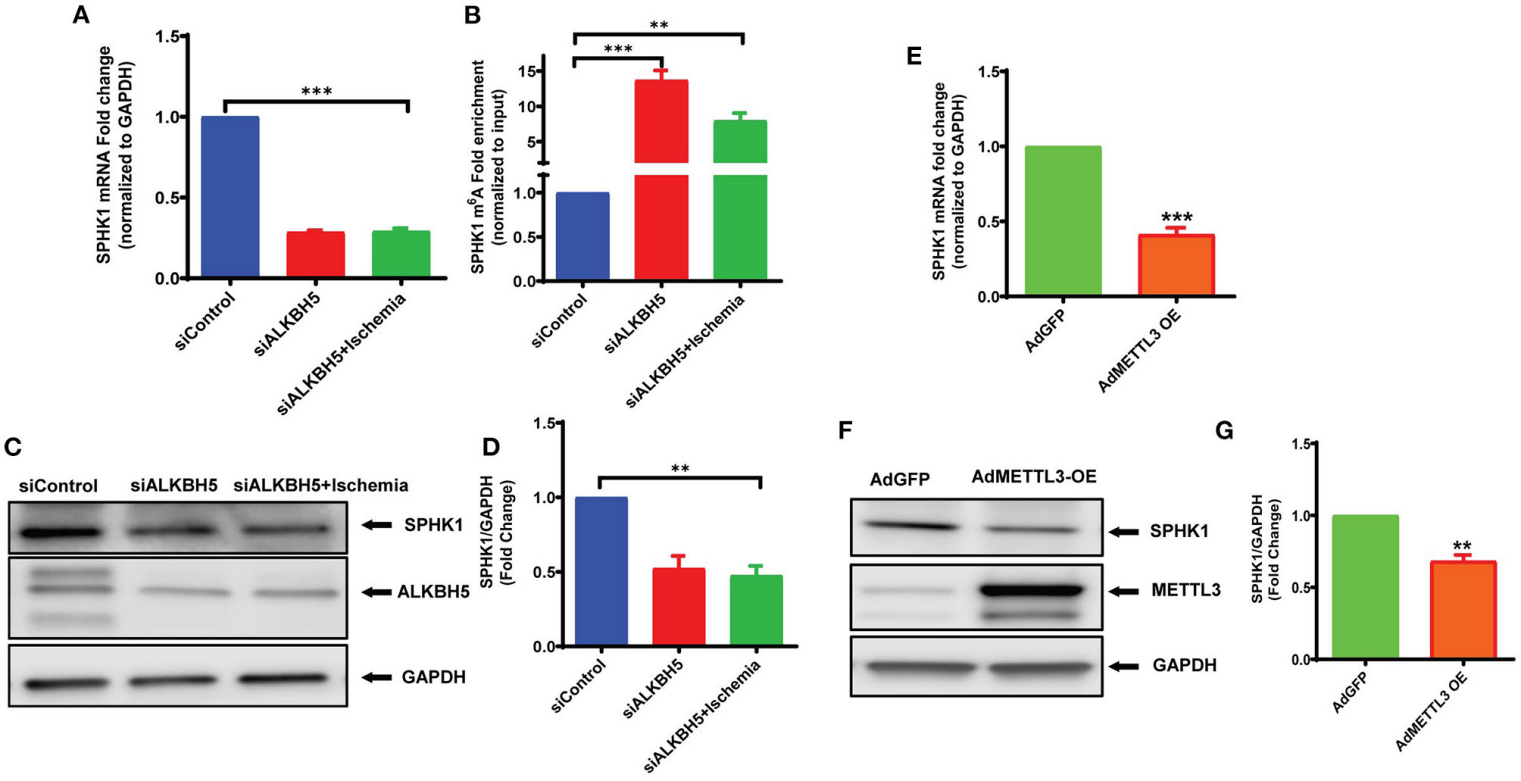
ALKBH5 silencing increased SPHK1 m^6^A mRNA methylation. mRNA was isolated for both RT-qPCR (no immunoprecipitation –nonIP) and MeRIP (m^6^A methyl RNA immunoprecipitation) followed by MeRIP-qPCR. **(A)** RT-qPCR for SPHK1 showing decreased mRNA expression. **(B)** MeRIP-qPCR showing the m^6^A abundance on SPHK1 mRNA under given conditions. As expected, m^6^A mRNA methylation of SPHK1 was increased after ALKBH5 siRNA knockdown. Data is presented as m^6^A fold enrichment after normalization with nonIP input. **(C)** WB and **(D)** quantification of SPHK1 protein after ALKBH5 silencing with and without ischemic stimulus. ALKBH5 silencing alone or ischemia significantly reduced SPHK1 expression. **(E–G)** METTL3 was overexpressed using METTL3 adenovirus. METTL3 overexpression significantly reduced both SPHK1 gene **(E)** and protein expression **(F,G)**. SPHK1 bands intensity was measured with ImageJ (NIH) software. **P < 0.01, ***P < 0.001, ns indicates not significant with respective control (*n* = 3–5).

To further understand the mechanism(s) of ALKBH5 in the regulation of EC angiogenesis, we performed pathway-based RT^2^ profiler array analysis of the functional endothelial genes to analyze the gene expression following stress. RT2-profiler array data showed that hypoxia significantly alters the expression of many genes including SPHK1 (Figure 5A). Previously, it has been shown that SPHK1 regulates eNOS phosphorylation which in turn regulates ECs angiogenesis (10, 44, 45). Thus, we measured SPHK1 levels following ischemic stress. The SPHK1 protein expression was significantly upregulated following ischemic stress (Figures 5B,C). To determine whether ALKBH5 regulates SPHK1 gene and protein expression, we inhibited ALKBH5 in ECs using siRNA. Surprisingly, both mRNA and protein levels of SPHK1 were significantly decreased after ALKBH5 silencing (Figures 6A,C,D). m^6^A mRNA methylation plays important role in stability/degradation of target RNA. Thus, we hypothesized that ALKBH5 inhibition enhanced SPHK1 mRNA methylation and its rapid degradation. To validate this hypothesis, methyl RNA immunoprecipitation (MeRIP) followed by qPCR were performed in mRNA isolated from ECs following ALKBH5 inhibition. Interestingly, MeRIP-qPCR data suggests that ALKBH5 inhibition significantly increased SPHK1 mRNA methylation (Figure 6B). These data suggest that high SPHK1 m^6^A methylation after ALKBH5 downregulation may lead to its faster degradation (Figures 6A,C,D). Further to prove that SPHK1 mRNA is the target of m^6^A mRNA methylation, we employed gain-of-function approach using adenovirus-mediated overexpression of METTL3, a global m^6^A methyltransferase. As shown in Figures 6E–G, overexpression of METTL3 (increased m^6^A mRNA methylation) significantly reduced SPHK1 transcript level as well as the protein (Figures 6E–G). Overall, our data confirm that ALKBH5 regulates SPHK1 m^6^A mRNA methylation and possibly its degradation.

### SPHK1 is Required for Endothelial Cell Tube Formation

**Figure 7:**
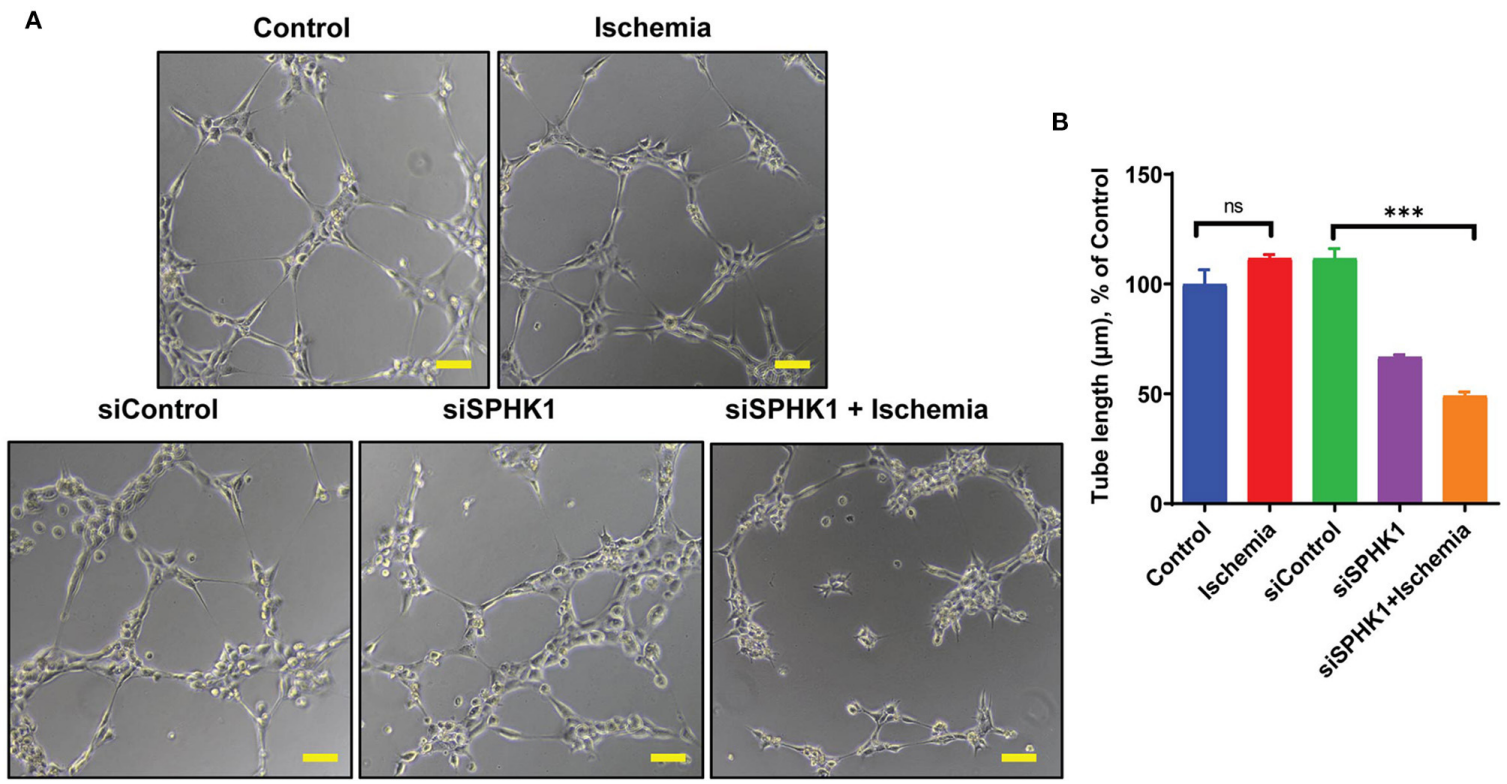
SPHK1 regulates endothelial cell tube formation under ischemic stress. **(A)** SPHK1 was downregulated using 30 nM siSPHK1 siRNA. For tube formation analysis, cells were trypsinized and seeded on matrigel. **(B)** ECs tubes length was observed under the microscope at 100X magnification and tube length was measured with ImageJ (NIH) software (around 50–70 branch points were measured to take the average for each specimen). The scale bar on Image represents 50 μM pixel size. The data are shown as means ± SEM using one-way ANOVA followed by Tukey's multiple comparison test. ***P < 0.001, ns indicates not significant with respective control (*n* = 3–5).

SPHK1 previously reported regulating EC functions including angiogenesis via eNOS and AKT phosphorylation (45). As per our data (Figures 2A,B), angiogenesis was preserved till 24 h following ischemic stress, which can be correlated with upregulated SPHK1 protein level (Figures 5B,C). Therefore, to study the role of SPHK1 in endothelial tube formation, we inhibited SPHK1 using siSPHK1 both in HMVE and HUVEC cells (Supplementary Figures 5A–D) before ischemia. EC tube formation was significantly impaired following SPHK1 inhibition. Our data suggest that SPHK1 is required for the maintenance of EC tube formation and its downregulation impaired this property (Figures 7A,B and Supplementary Figures 6A,B). Overall, this data confirms the role of SPHK1 in the maintenance of ECs tube formation.

## Discussion

Endothelial dysfunction plays important role in initiation and progression of coronary artery diseases. Therapeutic intervention targeting endothelial dysfunctions post-ischemic injury has been suggested to be an effective approach to regulate CVD (46–48). Endothelial dysfunction is commonly described by increased oxidative stress, reduced NO synthesis or availability and inflammation due to higher adhesion and migration of leukocytes (47, 49). The role of epigenetic modifications has been shown in the regulation of endothelial cell biology and functions. Mechanical stress such as disturbed blood flow caused DNA methylation, histone modification, and alteration in RNA-associated gene expression (50, 51). A growing body of evidence suggests that m^6^A mRNA methylation regulates hematopoietic and progenitor cell development from hemogenic ECs during mouse embryogenesis (52). In this paper, we showed that m^6^A mRNA methylation at *sphk1* mRNA (epitranscriptomic modification) leads to endothelial dysfunction following ischemic injury.

Generally, hypoxic microenvironment favors ECs-mediated angiogenesis and maintain an adequate oxygen and nutrients supply. In the adult heart, angiogenesis is induced by a number of factors including hypoxia, ischemia etc (53). In past decades, proangiogenic therapy to promote heart reperfusion and function appeared a promising strategy, however, so far, clinical trials have failed to meet the expectations. Therefore, the development of novel approaches to regulate or promote angiogenesis following ischemic injury is clinically relevant. In this study, we provide evidence, for the first time, that ALKBH5, a m^6^A demethylase, gets activated following ischemic stress and helps in the maintenance of tube formation. Furthermore, inhibition of ALKBH5 resulted in impaired EC tube formation following ischemia. Interestingly, the ischemia-induced eNOS phosphorylation (at Ser1177) was significantly reduced after ALKBH5 inhibition. Our data suggests that ALKBH5 regulates tube formation by regulating eNOS phosphorylation and its downstream signaling (54–58).

In heart and other organs several signaling mediators regulated eNOS phosphorylation (at Ser1177), including the phosphoinositide 3-kinase (PI3K)/AKT (59). Recently, it has also been shown that ALKBH5 regulates AKT phosphorylation and maintains bone morphogenetic protein ossification in the ligamentum flavum (60). Thus, it is possible that ALKBH5 may regulates eNOS phosphorylation and thus tube formation by maintaining AKT phosphorylation. Indeed, our results showed that inhibition of ALKBH5 downregulated the phosphorylation of both AKT and eNOS in ischemia. In contrast, VEGF-A protein level was unchanged following ALKBH5 silencing. VEGF-A and its receptors regulate angiogenesis by multiple pathways (61). Additionally, other members of VEGF family like placenta growth factor (PlGF), VEGF-C and VEGF-D as well as neuropilin and glial-derived neurotrophic factor (GDNF) are independent mediators of angiogenesis in endothelial cells (62–64). Furthermore, platelet-derived growth factor (PDGF), fibroblast growth factor and Delta/Notch signaling molecules also regulate VEFG-A independent angiogenesis (65–67). The data presented in this study showed a novel signaling mechanism, where ALKBH5 maintains EC angiogenesis independent of VEGF-A via eNOS phosphorylation following ischemic stress. Next, to understand ischemia-mediated gene regulation, we performed global gene expression analysis related to endothelial cell angiogenesis and found that hypoxic stress altered the expression of many key angiogenic genes including SPHK1 mRNA. Interestingly, we found a conflicting observation between SPHK1 mRNA and protein levels following ischemic stress. Previously, it has reported that transcript levels by themselves are not enough to predict protein levels in many scenarios such as during cellular differentiation or under stress condition, post-transcriptional processing (including m^6^A methylation) may lead to deviation from an ideal correlation (8).

ALKBH5 is a well-known m^6^A demethylase and plays a critical role in human diseases including heart disease. However, the role of ALKBH5 in the regulation of endothelial cell-mediated angiogenesis is not known. Intriguingly, we found an ALKBH5 dependent m^6^A mRNA methylation of SPHK1 transcript. Previous studies suggest that m^6^A mRNA methylation is selectively recognized by reader protein YTH domain family two (YTHDF2) which recruits m^6^A methylated transcripts to the mRNA decay sites and thus leads faster degradation of methylated RNA (18). In this study, our data suggest that AKLBH5 silencing under ischemic stress increased m^6^A level on SPHK1 mRNA and thus enhanced its degradation and reduced SPHK1 protein level. Furthermore, the reduced SPHK1 in ALKBH5 silenced cells may lead in decreased eNOS phosphorylation and ultimately tube formation.

SPHK1 is a known regulator of endothelial cell angiogenesis (45). Angiogenesis plays a critical role in the protection of myocardial tissue following ischemic injury. Early reperfusion of the occluded coronary artery has shown a substantial improvement in the outcome of MI patients by restoring blood supply to the infarcted area, hence reducing myocardial necrosis and fibrosis. Therefore, improvement of angiogenesis has the potential to salvage ischemic myocardium at the early stage after MI and is also essential for long-term myocardial remodeling to prevent the transition to heart failure. In past, many approaches have used to induce coronary collateralization (such as gene therapy, cell-based therapy etc) with limited success. The novel mechanism discussed in this paper to utilize epitranscriptomic approach to maintain or improve endothelial cell-mediated angiogenesis following ischemic injury is therapeutically significant. The data presented in this paper suggests that ischemia induced ALKBH5 activation reduce SPHK1 methylation and thus helps in the maintenance of tube formation. Previous studies suggests that SPHK1 regulates AKT phosphorylation and silencing of SPHK1 results in impaired AKT phosphorylation in renal cell carcinoma (44). Pharmacological inhibition of SPHK1 in hypertension and cardiac hypertrophy resulted in reduced eNOS phosphorylation (10). Also, PI3K/AKT regulates eNOS phosphorylation (59). Thus, elucidating SPHK1 mediated regulation of eNOS phosphorylation directly or *via* AKT could be interesting to understand the signaling interplay between ALKBH5, SPHK1, AKT and eNOS and their impact in regulation of EC angiogenesis. In conclusion, here for the first time we have reported that ALKBH5 mediated SPHK1 and eNOS signaling play important role in the maintenance of angiogenic potential in endothelial cells following ischemic stress.

## Materials and Methods

### Cell Culture and siRNA Transfection

Human umbilical vein endothelial cells (HUVEC) and human microvascular endothelial cells (HMVE) were obtained from ATCC (ATCC-CRL1730) and maintained in EBM-2-MV medium (Lonza, #CC3202) under 5% CO_2_ at 37 C for routine culture. To induce ischemic injury, cells were subjected to hypoxia (1% O_2_ at defined time points) with lipopolysaccharide (LPS, 100ng/ml) in EBM-2 media to mimic the inflammatory and hypoxic environment of ischemic heart and harvested for mRNA and protein isolation (68, 69). For efficient gene silencing, cells were transfected with siRNAs against *alkbh5* (10 nM; Dharmacon, M-004281-01-0005), *sphk1* (30nM; Dharmacon, L-004172-00-0005) and control siRNA (ThermoFisher Scientific AM4611) using transfection reagent from Mirusbio lab (TransIT-siQUEST, MIR2110). Transfection was done in complete EBM-2-MV having 5% fetal bovine serum (FBS) for 18–24 h. Following siRNA transfection, cells were treated with ischemic stress for described time-points and harvested for functional and biochemical analysis. For METTL3 overexpression, cells were infected with METTL3 adenovirus (vector lab) for 24 h before ischemic stress.

### Immunoblotting

To test the protein expression, HUVEC and HMVE cells were lysed with cell lysis buffer (Cell Signaling Technology (CST), #9803) supplemented with protease inhibitor cocktail (Thermo #78442). Proteins were separated by centrifugation at 12,000 g for 10 min at 4°*C* and protein concentration was determined using BCA protein assay kit (Thermo Fisher Scientific, USA). Total cellular proteins were separated in 4–15% SDS-PAGE, transferred on PVDF membrane followed by 1 h blocking in skim milk at room temperature (RT). Immunoblotting was performed using primary antibodies against ALKBH5 (Abcam, #195377), METTL3 (Abcam, # ab195352), AKT (CST, #2920), phospho-AKT (CST, #4060), eNOS (CST, #9572), phospho-eNOS (CST, #9571), VEGF-A (Abcam, #46154), SPHK1 (CST, #12071) and GAPDH (CST, #5174). All primary antibodies are used at 1:1000 dilutions, overnight at 4°*C*. Species-specific HRP-linked secondary antibodies were used at 1:2000 dilutions at RT for 1 h. Signals were detected using Odyssey® Fc Imaging System (LI-COR Biosciences). For quantitative and statistical analysis, the ImageJ (NIH) and GraphPad PRISM software was used, respectively.

### Tube Formation Assay

To examine the role of ischemic stress on tube formation of HUVEC and HMVE, cells were cultured either under normal or ischemic conditions for 4, 12, 24 and 48 h with or without respective inhibitors. Angiogenesis was assessed with tube formation assay as described previously with small modifications (70). Approximately, 15,000 cells were plated on matrigel (Corning, #CB-40230C) in 48 well plates for 6–8 h. Tubes were observed under a phase-contrast microscope (Nikon Eclipse Inverted Phase Contrast Microscope, Spectra Services) and imaged at 20X magnification, The bar scale on image represents pixel size of 50 μM length. The length of branch points was calculated using NIH ImageJ software and plotted (GraphPad PRISM software) to examine the tube formation efficiency under given conditions.

### Scratch Assay

HMVE/HUVEC cells were seeded and were transfected with scramble or siALKBH5 siRNA for 24 hrs. There after, a straight scratch was lined using a 200 μl tip in the fully confluent monolayer. Pictures were taken using a phase-contrast microscope (Nikon Eclipse Inverted Phase Contrast Microscope, Spectra Services) and imaged at 20X magnification. The bar scale on image represents pixel size of 50 μM length. This was considered as zero time-point. Further, cell were incubated under normal or ischemic conditions for 24 h and pictures were retaken. The cellular migration rate was assessed as the area covered with time and data were analyzed using NIH ImageJ software and plotted (GraphPad PRISM software).

### Terminal Deoxynucleotidyl Transferase–Mediated dUTP Nick End-Labeling Staining for Cell Death Analysis

HMVE cells were seeded on gelatin precoated coverslips in a 12-well plate and further cultured either under normal or ischemic condition with or without ALKBH5 silencing. Cells were washed with 1X PBS followed by fixation in 4% paraformaldehyde for 10 min. Cell death was analyzed using TUNEL staining kit (Cell death detection assay; Roche, Indianapolis, IN). CD31 was used as EC specific marker and DAPI was used to count the total number of nuclei. TUNEL with DAPI resulted in pink color. The percentage of apoptotic cells was calculated as TUNEL positive nuclei/total number of nuclei in the microscopic field.

### Analysis of Apoptotic Cell Death by Flow Cytometry

To study the cell apoptosis and its role in EC tube formation impairment, HMVE cells were cultured either under normal or ischemic condition with or without ALKBH5 silencing. Apoptotic cell death was determined by flow cytometry using the Alexa fluor 488 Annexin V Kit (# V13241, Invitrogen) following the manufacturer's protocol. Briefly, after siALKBH5 silencing and ischemic stress, around 0.5 million cells were harvested, washed with PBS buffer and incubated with Annexin V Alexa fluor488 (Alexa488) and propidium iodide for 30 min in the dark. The cells were then analyzed by fluorescence-activated cell sorting (FACS) using the FACS Attune NXT instrument and FlowJO software at the UAB Comprehensive Cancer Center core facility. One-way ANOVA (GraphPad Software Inc.) with *post-hoc* Tukey test was used to calculate statistical significance.

### mRNA Isolation

For all studies, mRNA was isolated using mRNA (NEB, S1550S) isolation kit according to manufacturer's protocol. 1 μg mRNA was used for m^6^A immunoprecipitation. For normal RT-qPCR, 100ng mRNA was used for cDNA synthesis.

### Gene Expression Analysis and Real-time qPCR

To study the changes in gene expression, 100 ng of total mRNA was reverse transcribed using TaqMan™ universal master mix II (ThermoFisher Scientific, #4440042) according to the manufacturer's protocol and amplified using Quantstudio3 (Applied Biosystem) with respective TaqMan assays from ThermoFisher Scientific. Gene expression levels of SPHK1 (#4331182, #Hs00184211_m1) was quantified from reverse amplified cDNA and normalized against GAPDH (#4331182, #Hs02786624_g1) expression as an internal control. The difference was represented as fold change using the ^Δ*ΔCT*^ method. One-way ANOVA was used for calculating statistical significance in GraphPad Prism.

### RT^2^ Profiler PCR Array Analysis

To analyze the expression of EC-specific genes under hypoxia, pathway-focused RT^2^ profiler PCR array from Qiagen (#- 330231 PAHS-015ZA) was used. First strand cDNA synthesis was achieved with 250 ng mRNA using RT^2^ First Strand Kit (Qiagen, #-330401). The genomic DNA elimination step was performed as described in the user manual. Further, RT^2^ SYBR green master mix (Qiagen, #-330500) was added to the diluted cDNA and the reaction was equally distributed into an array plate. Data analysis was done using Qiagen data analysis tools (https://geneglobe.qiagen.com/us/analyze).

### m^6^A Immunoprecipitation and RT-qPCR (MeRIP-qPCR)

To study the relative m^6^A levels on SPHK1 mRNA, mRNA was isolated from ECs after treatments. A 1/10 portion of mRNA was saved as the input aside to perform normal RT-qPCR, and the remainder mRNA was processed for RNA fragmentation (~100 nucleotides small fragments) using fragmentation buffer (buffer- 100 mM ZnCl_2_ in 100 mM Tris–HCl pH-7.0) by incubating at 94°C for 5 min. the reaction was stopped immediately by adding 50 mM EDTA. For immunoprecipitation (IP) of m^6^A methylated fragments, 1 ml of IP buffer was added in each reaction with 2 μg anti-m^6^A antibody (Epigentek, # A-1802-100) and incubated for 2 h at 4°*C* with continuous rocking (IP buffer: 50 mM Tris–HCl, 750 mM NaCl and 0.5% Triton-X100). Beads were washed twice with deionized water and equilibrated by adding 1 ml IP buffer having 0.5 μg/ml BSA with continuous rotation for 1:30 min at 4°*C*. After equilibration, beads were centrifuged and mixed with IP reaction buffer for 2 h with rotation at 4°C. Beads were washed three times with IP buffer each for 5 min and eluted using 250 μl elution buffer (150 mM NaCl, 50 mM Tris–HCl pH 7.5, 1 mM EDTA, 0.1% SDS, 20 mM DTT) at 42°*C* for 5 min. The supernatant was collected and m^6^A methylated mRNA was purified using RNeasy mini kit (Qiagen, # 74106) and eluted in 16 μl nuclease-free water. The methylated mRNA was used for cDNA synthesis for MeRIP-qPCR to test the m^6^A abundance on SPHK1 transcript.

### METTL3 Overexpression

METTL3 is the key m^6^A methyltransferase, we overexpressed METTL3 to study the m^6^A regulation of SPHK1. Approximately 80% confluent HUVEC cells were infected with adenovirus expressing either Ad-CMV-GFP (VECTOR BIOLABS, #-1060) alone (control) or Ad-GFP-m-METTL3 (VECTOR BIOLABS, ADV-264533) at MOI 1:10 and 1:20, respectively for 24 h. Cells were visualized under a fluorescent microscope for GFP expression and then harvested for both mRNA and protein isolation.

### Statistical Analysis

Results are expressed as the mean ± standard error of the mean (SEM), calculated from separate experiments. Comparison between control and experimental groups were performed using the unpaired *t*-test. Multiple groups comparisons were performed using one-way ANOVA (GraphPad Software Inc.) and levels of significance were determined with the Tukey-Kramer multiple comparison *post-hoc* test. The data presented in this manuscript are means ± SEM from 4 to 6 biological replicates. *p* < 0.05 were considered as statistical significance.

## Data Availability Statement

The raw data supporting the conclusions of this article will be made available by the authors, without undue reservation.

## Author Contributions

RK and SV: conceptualization. RK, RD, and SV: formal analysis. SV: funding acquisition. RK, RD, PR, ZS, JL, and HP: experimental procedure and data acquisition. SV: project administration, resources, and writing—review and editing. RK: writing—original draft. All authors contributed to the article and approved the submitted version.

## Funding

This research was funded by grant number HL135060 from National Institute of Health and 14SDG20480104 from American Heart Association to SV.

## Conflict of Interest

The authors declare that the research was conducted in the absence of any commercial or financial relationships that could be construed as a potential conflict of interest.

## Publisher's Note

All claims expressed in this article are solely those of the authors and do not necessarily represent those of their affiliated organizations, or those of the publisher, the editors and the reviewers. Any product that may be evaluated in this article, or claim that may be made by its manufacturer, is not guaranteed or endorsed by the publisher.
