# Supplementary material for: ALKBH5 Regulates SPHK1-Dependent Endothelial Cell Angiogenesis Following Ischemic Stress
Source: Front Cardiovasc Med. 2022 Jan 20;8:817304. doi: 10.3389/fcvm.2021.817304 (PMC8811170; doi:10.3389/fcvm.2021.817304)

Supplementary Material

Supplementary Figure 1. ALKBH5 silencing impaired the tube formation following ischemic stress in HUVEC cells. **(A-B)** HUVEC cells were co-treated with LPS with hypoxia in nutrient deficient media to induce ischemic stress for indicated time-points. **(C-D)** HUVEC cells were cultured under ischemic stress with or without ALKBH5 silencing for indicated time points. For tube formation analysis, cells were trypsinized and seeded on matrigel. Tube formation was observed under the microscope at 10X magnification and tube length was measured with ImageJ (NIH) software (total 40-50 branch points were measured to take the average for each specimen). One-way ANOVA followed by Tukey's multiple comparison test was used for calculating statistical significance. The data is shown as means ± SEM. *$P<0.05$, **$P<0.01$, ***$P<0.001$, ns indicates not significant with respective control.

Supplementary Figure S2. ALKBH5 downregulation significantly diminished EC migration after ischemic stress in HMVE cells. (A) HMVE cells were either transfected with scramble or siALKBH5 for 24 hrs and cultured under normal conditions. Wound was generated using a tip in fully confluent monolayer of HMVE and cells were further incubated under normal or indicated conditions. Wound healing was observed after 24 hrs and results were compared using One-way ANOVA. (B) Migration quantification. *** $P<0.0005$, siControl vs siALKBH5 with ischemic stress.

Supplementary Figure S3. ALKBH5 inhibition in **(A-B)** HMVE and **(C-D)** HUVEC cells. Both cells were transfected with either 10nM siRNA negative control or siALKBH5. ALKBH5 silencing was confirmed with WB. The student's t-test was used to calculate statistical significance. *$P<0.05$, ***$P<0.001$, with respective control. The same siRNA negative control and concentrations (10 nM for both negative and siALKBH5) were used throughout the study.

Supplementary Figure S4: Impaired tube formation following hypoxia and LPS co-treatment in ALKBH5 silenced cells is independent of cell death. **(A)** TUNEL assay was used to determine apoptotic HMVE cells. As shown in figure, immuno-staining was performed in 4% PFA fixed cells for CD31 (green) and TUNEL (red) as markers of endothelial cell and apoptosis, respectively, (100X, scale bar 100μm). DAPI was used to stain nucleus. Data suggested almost similar cell apoptosis in all given conditions. **(B)** The total number of TUNEL positive nuclei was divided with total DAPI stained nuclei. **(C)** HMVE cells were cultured with LPS+hypoxia with or without ALKBH5 downregulation.

Annexin V/PI staining was performed to quantitate the number of apoptotic cells using flow cytometry. Cell death was not affected by downregulation of ALKBH5. One-way ANOVA followed by Tukey's multiple comparison test was used for calculating statistical significance. ns indicates not significant.

Supplementary Figure S5. SPHK1 silencing in **(A)** HMVE and **(C)** HUVEC cells. Both cell types were transfected with either 30nM siRNA negative control or siSPHK1 siRNA. SPHK1 silencing was confirmed with WB. (B) and (D) Quantification. The student's t-test was used to calculate statistical significance. *P<0.05, **P<0.005, siRNA negative control vs 30 nM siSPHK1. The same siRNA negative control and concentrations (30 nM for both negative and siSPHK1) were used throughout the study.

Supplementary Figure S6. SPHK1 regulates endothelial cell tube formation following hypoxia and LPS co-treatment. **(A)** HMVE cells were cultured under LPS+hypoxia for 24 hrs with or without SPHK1 silencing. For tube formation, cells were trypsinized and seeded on matrigel. **(B)** Tube formation was observed under microscope at 10X magnification and tube length was measured with ImageJ (NIH) software (around 50-70 branch points were measured to take average for each specimen). The statistical significance was calculated using one-way ANOVA followed by Tukey's multiple comparison test. The data is shown as means ± SEM. **P<0.01, ***P<0.001, with respective control. ns indicates not significant.

Supplementary Fig S1

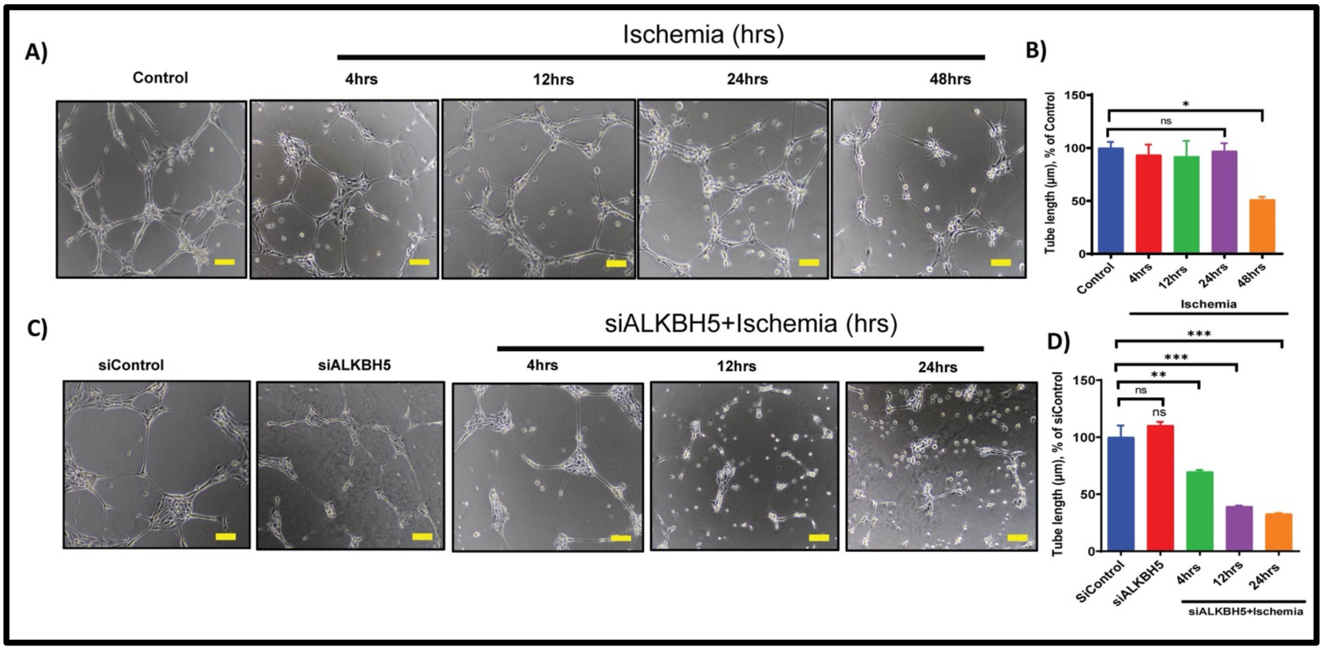

Supplementary Fig S2

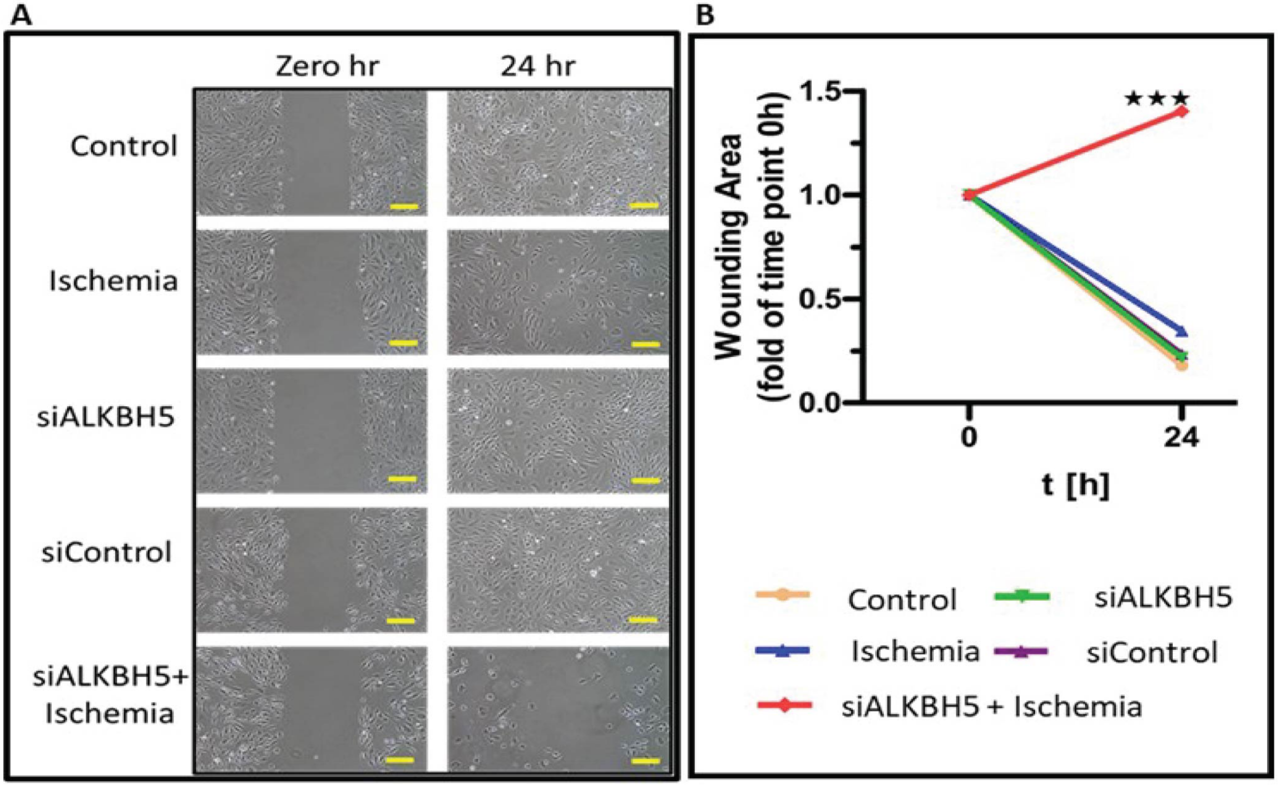

Supplementary Fig S3

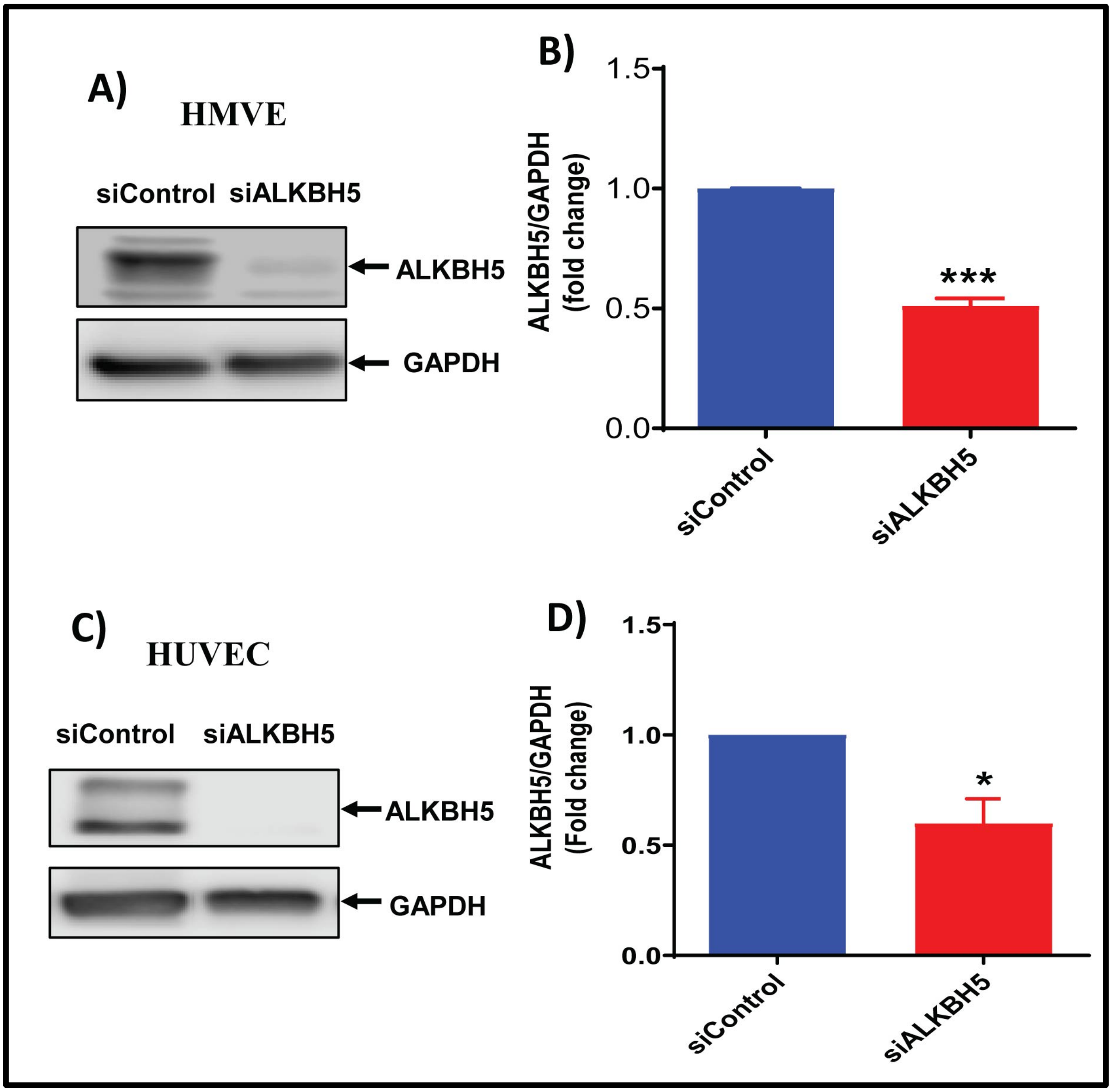

Supplementary Fig S4

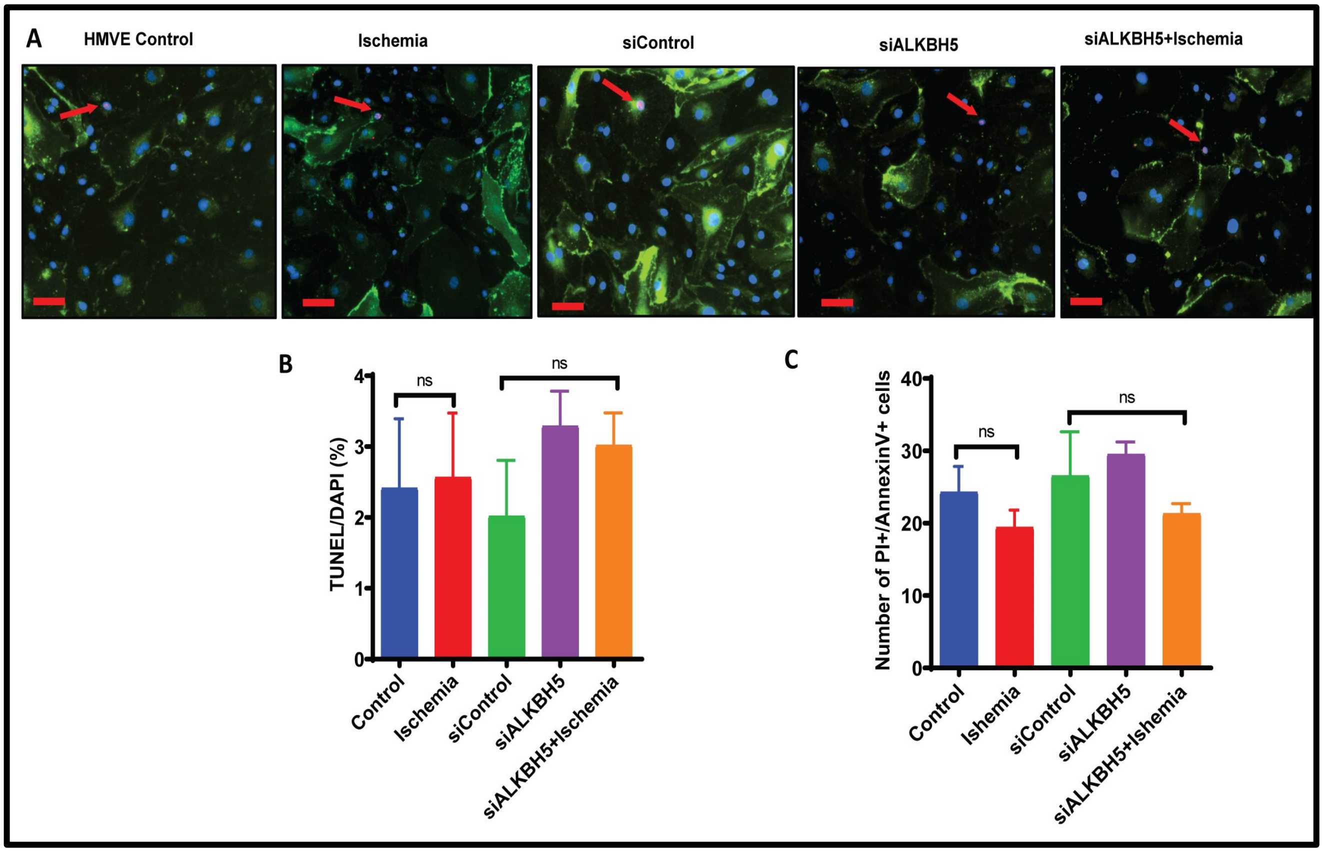

Supplementary Fig S5

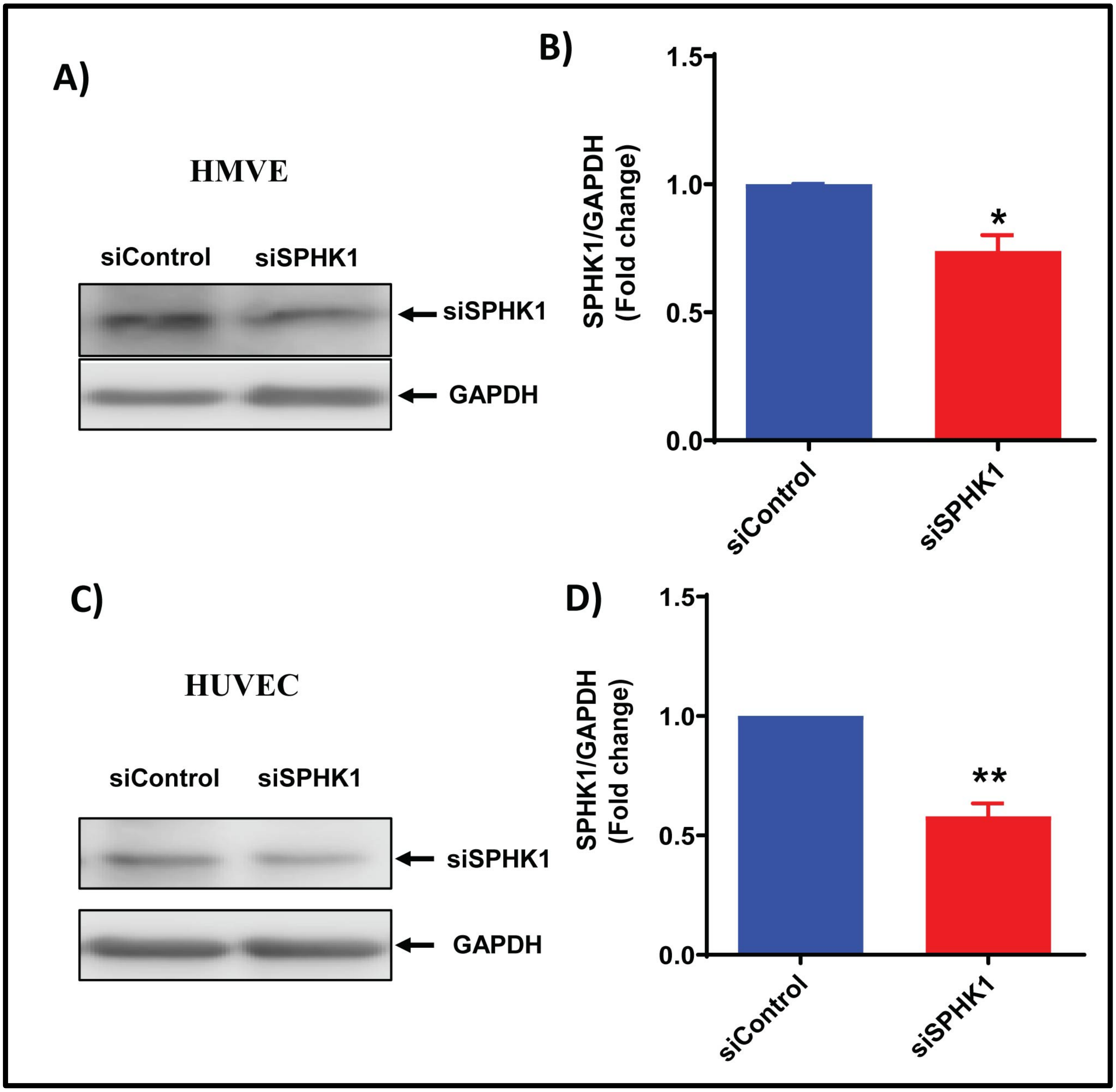

Supplementary Fig S6

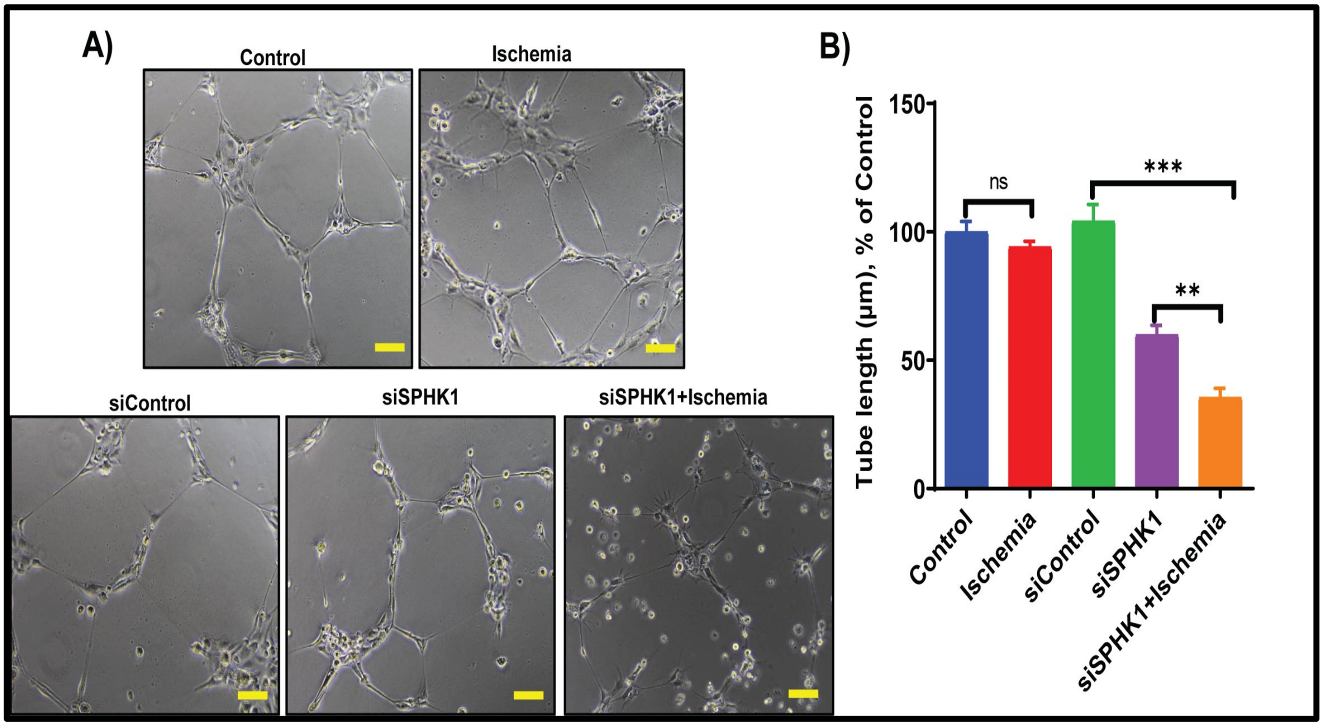

Supplement: Supplementary file 1 [file Data_Sheet_1.PDF]
